# Systematic review of heath care interventions to improve outcomes for women with disability and their family during pregnancy, birth and postnatal period

**DOI:** 10.1186/1471-2393-14-58

**Published:** 2014-02-05

**Authors:** Reem Malouf, Maggie Redshaw, Jennifer J Kurinczuk, Ron Gray

**Affiliations:** 1 Policy Research Unit in Maternal Health and Care, National Perinatal Epidemiology Unit, Nuffield Department of Population Health, University of Oxford, Old Road Campus, Headington, Oxford OX3 7LF, UK

**Keywords:** Disability, Pregnancy, Postnatal, Maternity, Systematic review

## Abstract

**Background:**

Health care providers are often unfamiliar with the needs of women with disability. Moreover maternity and postnatal services may not be specifically tailored to the needs of women with disability and their families. We conducted a systematic review to determine the effectiveness of healthcare interventions to improve outcomes for pregnant and postnatal women with disability and for their families.

**Methods:**

Studies on pregnant and postnatal women with disability and their families which evaluated the effectiveness of an intervention using a design that met the criteria used by the Cochrane Effective Practice and Organization of Care group were eligible for inclusion in this review. A comprehensive search strategy was carried using eleven electronic databases. No restriction on date or language was applied. Included studies were assessed for quality and their results summarized and tabulated.

**Results:**

Only three studies fully met the inclusion criteria. All were published after 1990, and conducted as small single-centre randomized controlled trials. The studies were heterogeneous and not comparable. Therefore the main finding of this review was the lack of published research on the effectiveness of healthcare interventions to improve outcomes for pregnant women with disability and their families.

**Conclusions:**

More research is required to evaluate healthcare interventions to improve outcomes for pregnant women with disability and their families.

## Background

More than 1 billion people, 15% of the global population, are disabled and a worldwide disability prevalence of 10% has been estimated among women of childbearing age [1]. The prevalence of self-reported disability among women of childbearing age has been reported as 11.7% in the USA [2] and, in the UK, estimates of long-term limiting illness in women who had recently given birth indicate a prevalence of 9.4% [3].

Disability is not a straightforward term to define and many definitions are in place. Therefore, the International Classification of Functioning Disability and Health (ICF) definition was adopted for conducting this review. The ICF states that: “Disability is a decrement in functioning at the body, individual or societal level that arises when an individual with a health condition encounters barriers in the environment” and “Disability is a complex phenomenon, reflecting an interaction between features of a person’s body and features of the society in which he or she lives” [1]. This dual nature of the concept of disability places as much emphasis on the environmental response to a health condition as it does on the health condition itself. This approach is further reinforced by the United Nations Convention on the Rights of Persons with Disabilities [4]. The particular impairments leading to restrictions on activity or participation in society are many and varied. For this reason, in this review we have considered disabilities of the following kinds: those related to impairments arising from physical disorders; those stemming from mental disorders; those associated with learning disabilities; and sensory and intellectual impairments.

The United Nations Convention enshrines human rights to education, employment, housing and transport, and in Article 23 promotes respect for home and family, specifically reproductive rights [5]. Yet women with disability are still confronted with considerable difficulties in pregnancy and childbirth. Many women with disability want to have children and are capable of conceiving, but face considerable pressure not to do so [6]. If they become pregnant and give birth, they may face negative attitudes and scepticism regarding their ability to parent effectively [7], particularly if they have intellectual disabilities [8]. However, a second national survey by the National Disability Authority in Ireland (NDA) revealed that public attitudes towards disability are changing [9] when compared to results from a 2001 survey [10]. Results showed more positive attitudes, to those with physical disabilities in relation to employment, education and accessing services. However, 41% to 84% of the respondents agreed that people with disability had the right to have children. However, a less positive attitude was held towards those with a mental health disability. Findings from a 2011 survey in Ireland showed some hardening of attitudes across all types of disability; 37%-69% of the public felt that people with disability could have children if they wish. The lower level of agreement was for people with intellectual disability and the highest was for people with physical disability [11].

Increasing numbers of women with disabilities wish to become mothers [12]. In one study of 144 women with spinal cord injury (SCI), 44% desired pregnancy and 36% conceived after the injury [13]. In both the United Nations Conventions on the Rights of Persons with Disabilities [5] and the UK Equality Act 2010, access to healthcare services is an important element [14]. It is the responsibility of service providers to make reasonable adjustments to ensure that health services are accessible. However, health care providers are often unfamiliar with the needs of pregnant women with disability, health professionals may be uninformed or even discriminatory, and services are often not specifically tailored to the needs of women with disability [15,16].

Although there is information about the reproductive experience of women with disability, and potential barriers to access as well as suggestions on how to improve services [17], there has been a dearth of evidence-based guidance on what works in improving services for this group. In addition, despite an extensive literature on the management of particular health conditions often associated with disability in a maternity context (such as epilepsy, mental health conditions, low back pain etc.), there seems to be little known about the management of disability more generally; that is, actions targeting disabling barriers rather than the health condition associated with disability.

The environments and policies within maternity services could either contribute to, or alleviate, disability. Therefore, we decided to undertake a systematic review with the objective of determining the effectiveness of healthcare interventions in improving outcomes for pregnant and postnatal women with disability and for their families. Any strategy aiming to change health care performance in this population was considered in this review. For example assistive technology interventions such as videos, telephone helplines, resource networks, parent-to-parent links. This is a broad aim and antenatal interventions were considered separately from postnatal interventions.

## Methods

A preliminary protocol was designed and then independently peer-reviewed. This outlined the literature search strategy, the selection process for identifying relevant studies, the method of extracting data from eligible studies, assessing the methodological quality of individual studies, and methods of data synthesis. A project advisory group, consisting of methodologists, clinicians and representatives of relevant users and voluntary groups working in the field of maternity care and disability were invited to comment on the protocol.

### Criteria for considering studies for this review

#### Types of studies

We only considered studies that met the criteria used by the Cochrane Effective Practice and Organization of Care group (EPOC) [18] for inclusion in this review. We felt that this was the most appropriate way of ensuring we were incorporating the best available evidence to answer the review question. The EPOC study designs are: Randomized Controlled Trials (RCTs), clustered Randomized Controlled Trials (c-RCT), Controlled Clinical Trials (CCT), Quasi-experimental studies, and Controlled Before and After trials (CBAs): studies were included if they had at least one intervention and one comparison group. Studies without a comparison group were not included.

#### Types of participants

We included studies carried out on the following groups: women with disability during the antenatal, intrapartum periods or in the postnatal period (up to 12 months following birth); families, partners and children of women with disability during the antenatal, intrapartum or postnatal periods; health care professionals at any time-point but aimed at improving quality and/or accessibility of maternity services for women with disability.

#### Types of interventions

Any intervention aiming to change health care outcomes for disabled pregnant and postnatal women and their families where the intervention took place in the antenatal period, during childbirth and in the first postnatal year was considered for inclusion. We only considered studies of interventions which were intended to tackle disabling barriers rather than the health conditions associated with disability. Studies focusing on treatment of underlying health conditions (where the focus was not primarily on the limiting or disabling aspects of these conditions) were excluded. So, for example, studies concerned with the clinical management of pre-eclampsia, anaemia, gestational diabetes, perinatal depression, and pelvic pain (all potentially disabling conditions) were not included in this review. For most of these conditions, the Cochrane Pregnancy and Child Birth Group [19] has already produced systematic reviews comparing effectiveness of different healthcare interventions. Interventions could be implemented in any setting: health care based setting (primary health care and hospital), home environment setting, and community setting.

#### Types of outcome measures

As we were not clear how many studies we would find or what outcomes would be used we considered all outcomes relevant to the health and social care of our population of interest with no specific exclusions. These outcomes could include preterm birth, birth weight, stillbirth, and mode of delivery; accessing health care services; and children-parent separation rate.

### Search methods for identification of studies

#### Searches

A comprehensive search strategy was developed (the MEDLINE search strategy is detailed in Additional file 1). Sets of search terms were developed to cover the following concepts: pregnancy, disability, health services and interventions. For the term disability, both categorical (for example: physical disability) and functional definitions (for example: spinal cord injuries), were used. The methodological component of the Cochrane Effective Practice and Organization of Care group (EPOC) search strategy was combined with selected index terms keywords and free text terms such as:

“Pregnant”, disabil*, disability, physical disability, decreased mobility, immobility, spinal cord injuries, head injuries, paralysis, paraplegia, quadriplegia, backache, disabled person, visually impaired person, vision disorders, blind, deaf, hearing impaired, hard of hearing, intellectual disability, learning disability, learning disab*, intellectual disab*, intellectual impair*, development* disab*, development* impair*, mental* retard*, mental* challenged, mental* handicap*, mental* impair*, mental* deficen*, subaverage intelligen*, mental* subnormal*, learning difficult*developmental disability; mental retardation, mental health, mental illness, severe mental illness, pre/postnatal depression, anxiety, schizophrenia, psychosis, bipolar disorder, eating disorders, multiple sclerosis, epilepsy, cerebral palsy, headache, muscular dystrophy, restricted growth/skeletal dysplasia, phocomelia, thalidomide survivors, myasthenia gravis, health services, perinatal care, postnatal care, preconception care, maternity services, mental health services, and health care.

We searched the following electronic databases for primary studies: The Cochrane Central Register of Controlled Trials (CENTRAL) (Issue 2, 2012); Medline, Ovid (1967 to March 2012); EMBASE, Ovid (1946 to March 2012); CINAHL, EBSCO (1980 to March 2012); PsychINFO, Ovid (1945 to March 2012); Latin American Caribbean Health Sciences Literature (LILACS); Index to Theses (1974 to March 2012); British Nursing Index (BNI) (1994 to March 2012); Social Science Citation Index (1952 to March 2012); and Sociological Abstract, CSA (1952 to March 2012). (See Additional file 2 for search report).

Web-based searches of disability and health services included the World Health Organization (WHO), The United Nations (UN), the Department of Health England (DH) and National Institute for Health and Clinical Excellence (NICE) websites. The reference lists of all included papers were searched. No language or date restrictions were applied to the searches. Attempts were made to obtain translations of included non-English articles.

### Data collection and analysis

#### Selection of studies

Articles were only rejected on initial screen if the reviewer could determine from the title and abstract that the article was not a report of an intervention, that the intervention did not address disability, or if the intervention was not conducted in relation to the antenatal or intrapartum period or in the first postnatal year. When a title/abstract could not be rejected with certainty, the full text of the article was obtained for further evaluation.

Following an initial screen to exclude non-relevant studies, potentially relevant studies were assessed independently for inclusion by two assessors (RM and RG) and differences between reviewers' results resolved by discussion and, when necessary, in consultation with a third reviewer. Studies were categorised as “included” or “excluded”.

### Data extraction and management

#### Data synthesis

A data extraction form was developed and included the following sections: study design, characteristics of the study participants such as age, disability, gestation at birth, intervention types, frequency and duration of the intervention, outcomes, and reported results. The data extraction form is available from the authors on request.

Data were extracted from published reports. When missing data were identified, investigators were approached. The data were then added to the data extraction form which contained data reported in the original paper and data ultimately collected by the reviewers. One reviewer extracted the relevant data (RM) and another reviewer (RG) checked this independently. Contradictions were resolved through discussion.

#### Assessment of risk of bias in included studies

Risk of bias was assessed independently by two reviewers (RM and RG). The eight EPOC-described criteria have been followed: sequence generation, allocation concealments, blinding, incomplete outcome data, selective reporting, baseline characteristics, baseline outcomes, protection against contamination and other bias. Each criterion was given one of the following ratings: “yes”, “no” or “unclear”. Any discrepancies in quality rating were resolved by discussion.

#### Data synthesis

As we expected, the studies we identified were of an extremely heterogeneous nature. For this reason we predominantly used tabulation and narrative synthesis in summarizing the data.

## Results

### Results of the search

**Figure 1:**
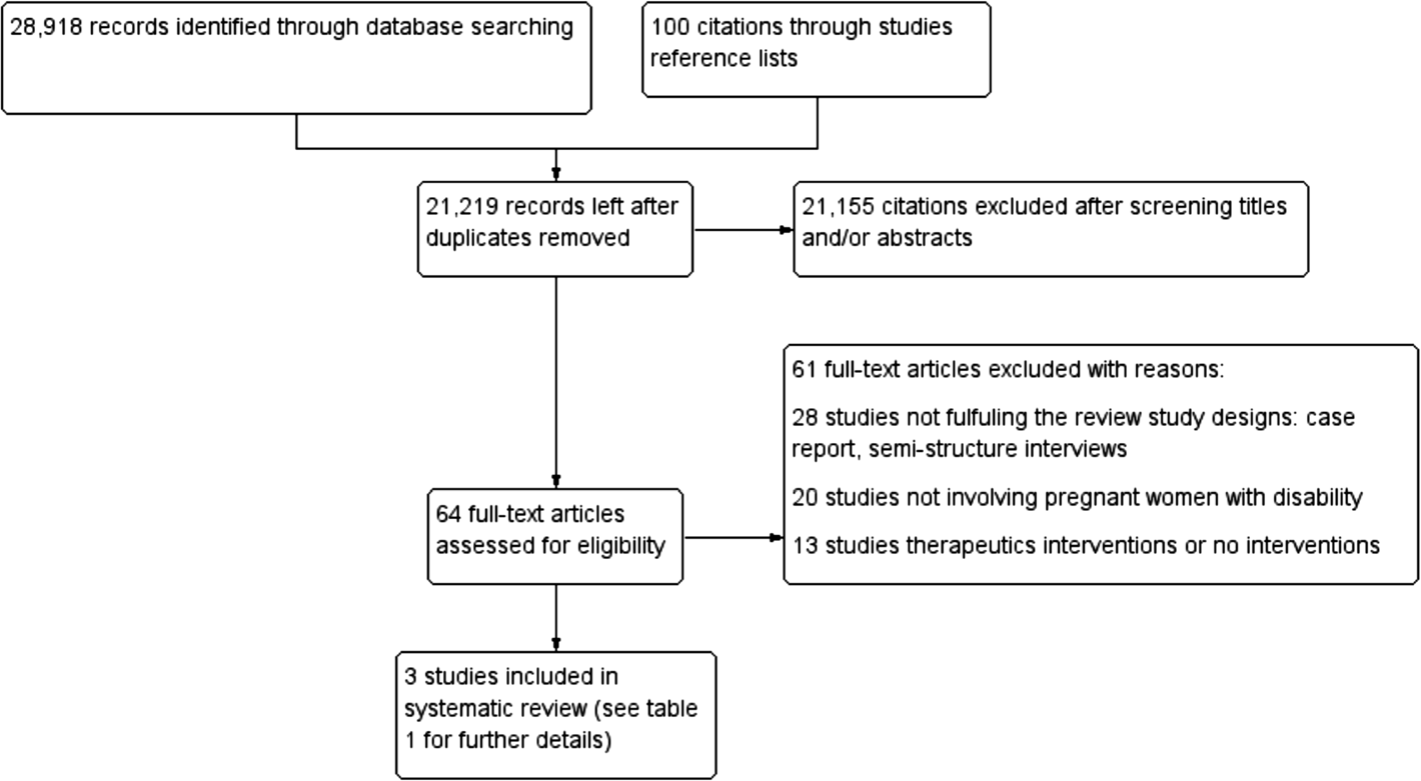
Flow diagram for study retrieval and selection.

The electronic search strategy yielded 28,918 citations and a hundred references from other sources (Figure 1). Of these 7,799 were duplicates either within the same data base or across data bases. Following screening titles and abstracts (when present), 21,155 were excluded. Sixty four citations were identified as potentially relevant. Examination of the full text resulted in the exclusion of 61 studies. Additional file 3 presents list of references and the reasons for excluding these studies.

### Description of included studies

Only three studies fully met the eligibility criteria and were included in the review. They were all single-centre RCTs (Keltner et al., Barbosa Regia, Scafidi & Field) [20-22]. Two studies were conducted in a hospital setting (Barbosa Regia and Scafidi & Field) [21,22] and one in a community setting (Keltner et al.) [20]. Two studies were conducted in the United States- (Keltner et al. and Scafidi & Field) [20,22], and one in Brazil (Barbosa Regia) [21]. One study lasted up to one year (Keltner et al.) [20] and one (Scafidi & Field) [22] for ten days and in the case of (Barbosa Regia) [21], 48 hours. Two studies were published in English and one in Portuguese. The studies involved a small number of participants and only covered two groups of women with disability, those with physical disabilities and those with “developmental disability” (sic). Attempts to obtain additional data from two studies (Keltner et al.) [20] and (Barbosa Regia) [21] met with no response. The characteristics of the included studies are outlined in Table 1.

**Table 1:** Characteristics of the included studies

**Study**	**Participants**	**Intervention**	**Outcomes**	**Results**
**Keltner**[20]	Inclusion criteria:	Intervention group: Supports to Access Rural Services (STARS), met weekly with a family service worker for one year. This has 3 main domains: Staff training (learning about disability, recognizing health and social disorders, crisis intervention, cultural sensitivity, community liaison skills and maintaining realistic expectations); STARS activities to support mothers' self-esteem and other family members; case co-ordination: to identify the families' needs earlier and to provide the related support to access services.	Nursing Child Assessment Teaching Scale (NCATS) (Barnard [26]): It has six sub scales, four of which focus on the parents and two on the child.	NCATS means subscales scores changes after one year:
RCT, USA, community setting	40 women who had children age between 12 and 36 months; maternal IQ < 85; low income families.			
			Parent’s subs-sales domains are: child's cues, responsiveness to distress, social-emotional interaction, and cognition growth fostering.	For mother's sensitivity to child's cues: 1.1 in the STARS group compared with 0.1 in the control.
	Intervention group: Mean age 25.4 years; mean IQ = 59; mean maternal years of education 10.5 years; mean number of children 2.2; mean child age 24.5 months; 46% were married.			
	Control group: Mean maternal age 22.6 years; mean = IQ 62.6; mean maternal years of education 11.5 years; mean number of children 1.8; mean child age 27.8 months; 31% were married			
		Control group: received a monthly contact by telephone, in-person assessments every 6 months.	Child sub scales are: clarity of cues and responsiveness to parents. Scoring is by the number of yes out of the 73 items.	Mother's responsiveness to distress: 2 in the STARS group compared with 0.1 in the control.
			Follow-up points: baseline, 6 months, 12 months.	Mother's social-emotional growth fostering: 1.2 for the STARS group compared with -0.1 in the control.
				Cognitive growth fostering: 2.2 in the STARS group and 1.0 in the control group.
				Child's clarity of cues: 0.4 in the STARS group compared with -0.6 in the control and on child.
				Child’s responsiveness to parents: 1.6 in the STARS group compared with -0.2 in the control.
				Mother-child interaction at 12 months: 8.3 in the STARS and 0.4 in the control, p < 0.05.
**Scafidi**[22]	Inclusion criteria:	Massage group:	1) Brazelton Neonatal Behaviour Assessment scale (Brazelton [27]): it is neuro-behavioural assessments to newborn’s abilities. The scale consists of 28 behavioural items scored on a nine-point scale and 18 elicited reflexes scoring on a three points scale.	Brazelton score(mean, Sd) massage therapy vs. control at day 10:
RCT, USA, hospital setting	HIV-exposed babies; delivered vaginally; average gestation age 39 weeks.	3×15-minute periods during three consecutive hours every day for 10 days (Monday to Friday). First session was begun within 30 minutes following the noon feeding, the second scheduled in 45 minutes after the completion of the first session, and the third session was within 45 minutes after the completion of the second session.		
				Habituation: 6.8 (0.4) versus 4.6 (0.5), p = 0.01.
				Orientation: 4.5 (0.3) versus 4.4 (0.5), p > 0.05.
	Exclusion criteria:			
	Babies with chromosomal aberrations; congenital heart malformations; infections: meningitis, herpes encephalitis; ventilatory assistance, medically unstable; receiving intravenous medications or feedings were excluded.			
	A 28 singleton neonates identified as HIV-exposed. Women were 67% African American and 33% Hispanic.			
			2) Weight gain	Motor: massage therapy group 5.2 (0.5) versus 4.5 (0.4), p = 0.001.
				Range of state: 4.3 (0.4) vs. 3.6(0.3), p = 0.05.
		Control group:		
				Regulation of states: 4 (0.6) vs. 4.5(0.7), p > 0.05.
		No massage		
		Both received standard care		Autonomic stability: 6.2 (0.7) versus 5 (0.5), p = 0.003.
		Both groups received a low number of visits by their parents (mean =4 in 10 days).	Follow-up points: at day 1 and day 10	Reflexes: 2.2 (0.3) versus 2.7 (0.2), P > 0.05.
				Excitability: 1.5 (0.4) versus 3.2 (0.4), p = 0.01.
				Depression: 3(0.4) versus 2.9 (0.4), P > 0.05.
				Stress behaviours: 1.8 (0.2) versus 3.6 (0.5), p = 0.004.
				Weight gain: 33.4 (4.3) versus 26.3 (3.9), p = 0.01.
**Barbosa Regia**[21]	24 HIV-positive pregnant women and their newborn babies, age range between 19–44 years, 17 (71%) were not married, 1 (4.2%) was a widow, and 6 (25%) were single. Only 58% had completed primary schooling, 25% had planned their pregnancy.	Intervention group (n = 12): An educational video to promote attachment between mothers and their newborns provided. The video was demonstrated by trained nurses in the prenatal period.	The 0–6 month Mother-baby Interaction Observation scale (Schermann [24]): It is a direct observation assessment of the behaviours between mothers and babies. It contains 21 items, 12 are related to mother’s behaviour and 8 to baby’s, 1 item for the mutual interaction.	Behaviours comparison between video and control groups for all five degrees of reaction from low to constant (KS*, p < 0.05):
RCT, Brazil, hospital setting				
				Verbal communication with the baby (KS = 1.255, p = 0.1).
				Eye contact (KS = 1.837, p = 0.002).
				The amount of positive affect (KS = 2.44, p = 0.0).
		Control group (n = 12): no intervention	Domains related to eye contact, attention to the baby, reactions to the child’s crying and sensitivity and physical contact with babies were observed in the study and rated as: None, low, moderate, maximum and constant reaction at 48 hours after delivery.	Mother’s attention to baby (KS = 1.255, p =0.0).
				Sensitivity comparison (KS = 1.837, p = 0.002).
				Comforting the babies when they cried (KS = 1.414, p = 0.037).
				Reaction to crying (KS = 1.414, p = 0.037.
				Response intensity (KS = 1.837, p = 0.002).

*KS = Kolmogorov-Smirnov statistical test.

### Methodological quality of included studies

**Figure 2:**
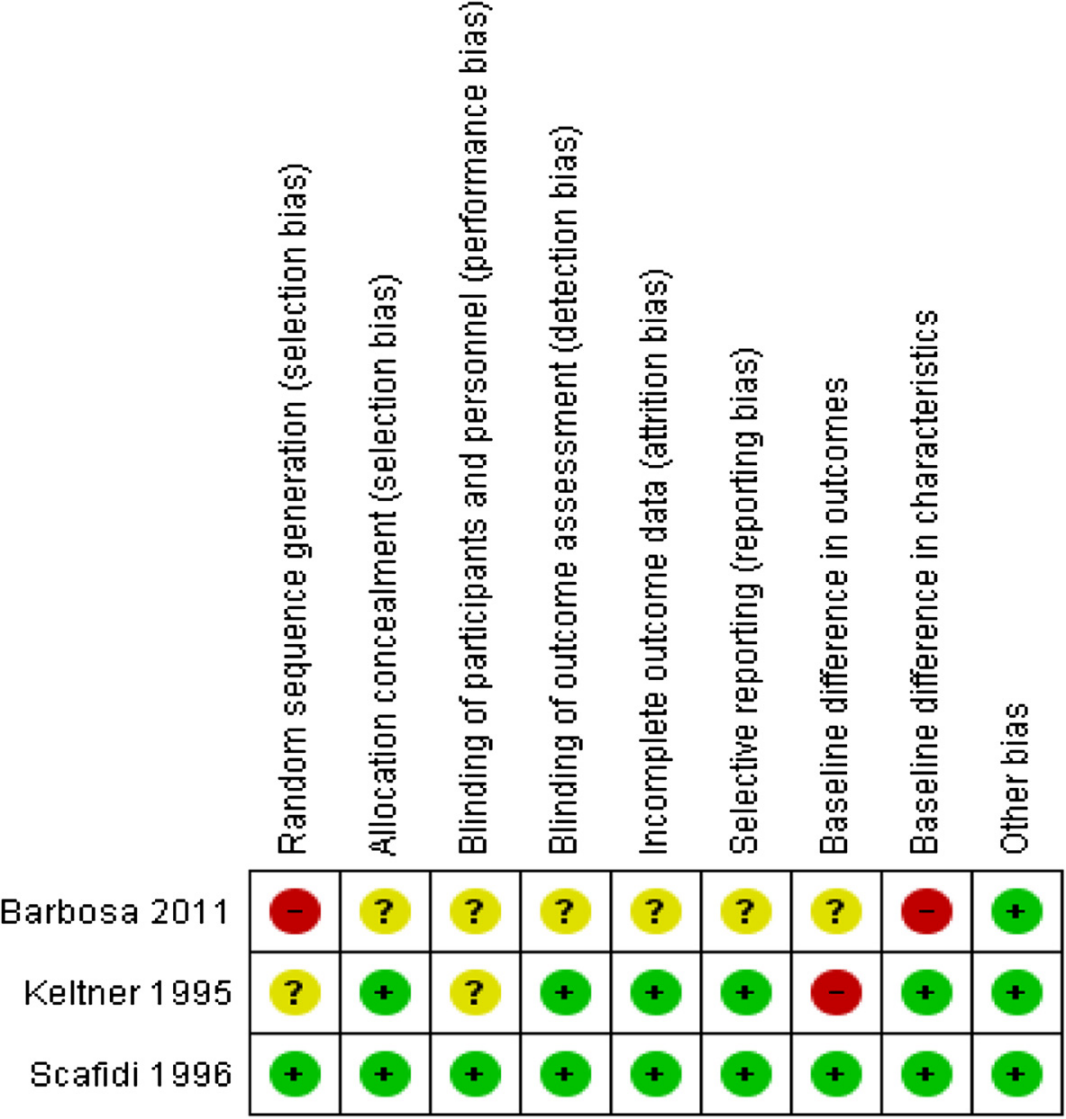
Summary of risk of bias.

**Figure 3:**
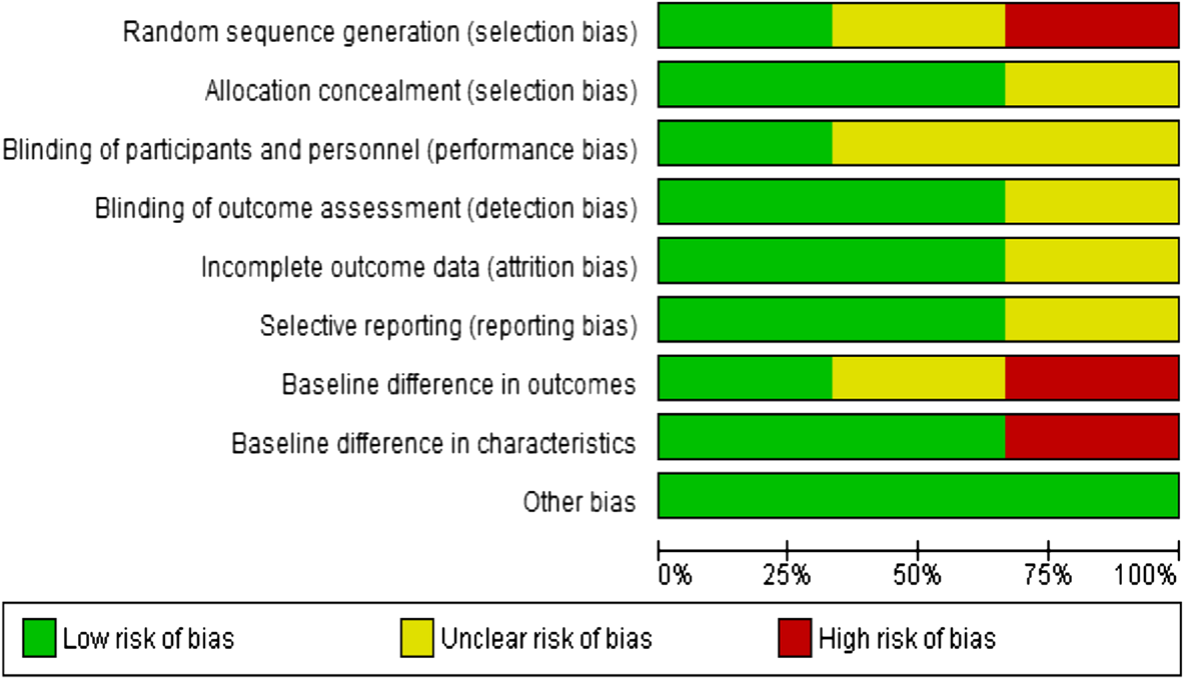
Risk of bias graph.

One study was rated as good quality (Scafidi & Field) [22], one of medium (Keltner et al.) [20] and one of low quality (Barbosa Regia) [21] with limited information available for some domains (see Table 2, Figure 2 and Figure 3). Studies were classified according to the level of allocation concealment: low risk of bias (adequate allocation concealment); moderate risk of bias (unclear allocation concealment) or high risk of bias (inadequate allocation concealment) [23]. The allocation concealment methods were provided in two studies (Keltner et al., Scafidi & Field) [20,22] (60%), and were performed by a person not associated with the project. In Barbosa [21] the allocation concealment method was unclear (see Figure 3). Adequate methods of sequence generation were reported in both Barbosa Regia and Scafidi & Field [21,22]; this was unclear in Keltner et al. [20]. In Scafidi & Field [22], a table of random numbers was used. However, in Barbosa [21] weekly alternation was set up, with one week for the tested group and the following week for the control.

**Table 2:** Risk of bias of the included studies


**Keltner**[20]	**Author’s judgment**	**Support of judgment**
Allocation sequence (potential selection bias)	Unclear risk	Method not specified
Allocation concealment (potential selection bias)	Low risk	Allocation was performed independently by a person not associated with the project
Baseline differences in outcomes	High risk	Imbalanced for the marital status and not adjusted
Baseline differences in characteristics	Low risk	No significant difference see Table 1
Incomplete outcome data (potential attrition bias)	Low risk	No attrition
Knowledge of interventions (potential detection bias)	Low risk	Assessed blindly: assessors blinding: local professionals who would likely know some or all of the families participating were not involved in the assessments
Contamination	-	-
Selective outcome reporting	Low risk	No other outcomes mentioned
Other risks of bias	Low risk	
**Scafidi**[22]	**Author’s judgment**	**Support of judgment**
Allocation sequence	Low risk	Random number table
Allocation concealment	Low risk	Randomised by researcher not associated with the project
Baseline differences in outcomes	Low risk	Imbalanced but adjusted
Baseline differences in characteristics	Low risk	Adjusted
Incomplete outcome data	Low risk	No drop out
Knowledge of interventions	Low risk	Assessors were blind to the group intervention
Contamination	-	-
Selective outcome reporting	Low risk	No other outcomes mentioned
Other risks of bias	Low risk	
**Barbosa**[21]	**Author’s judgment**	**Support of judgment**
Allocation sequence	High risk	By clinic attendance day
Allocation concealment	Unclear risk	No information
Baseline differences in outcomes	Unclear risk	No information
Baseline differences in characteristics	High risk	No comparison made between the groups
Incomplete outcome data	Unclear	No information
Knowledge of interventions	Unclear	No information
Contamination	-	-
Selective outcome reporting	Low risk	Only reported items measured
Other risks of bias	Low risk	

Blinding of participants was possible in Scafidi & Field [22], as the study enrolled new born babies. In Keltner et al. [20], it was unclear if the participants were aware of their intervention status. Blinding of outcome assessors was also reported in two studies (60%). In Scafidi & Field [22], examiners blind to group membership conducted the assessments in a small quiet room close to the nursery. In Keltner et al. [20], the assessments were carried out by a trained healthcare worker who was blind to the group assignment. However, blinding of participants and performance was unclear in Barbosa Regia [21]. Where blinding of participants and personnel was not possible or unclear, but blinding of outcome assessors was reported, the quality of evidence was not downgraded.

Two of the studies were free of risk of incomplete outcome data (attrition bias), this was not clear in Barbosa Regia [21]. Baseline characteristics were reported for both groups in Keltner et al. and Scafidi & Field [20,22] studies with no significant differences found. No information on this was reported in Barbosa Regia [21].

Imbalance in the marital status of the participants was mentioned in Keltner et al. [20], where 50% were married in the intervention group compared to a third in the control. No adjustment in the analysis was undertaken.

The studies were free from selective outcome reporting and other risk of bias.

### Results of included studies

It was not possible to perform a meta-analysis as the included studies were not sufficiently homogeneous. Studies varied in the categories of participants, the types of intervention and the outcomes measures applied.

Interventions to improve quality and accessibility of prenatal care for women with disability and their families: One study was included in this review which tested the effects of an intervention in the prenatal period. Barbosa Regia [21] investigated the effect of an educational video to promote mother-child attachments between HIV-positive women and their new-born babies. The educational video to promote the attachment between the mother and newborn baby was provided for women in the intervention group (n = 12). No intervention was reported for the controls (n = 12). The level of interaction between the mother and her baby was observed and the maternal behaviours were recorded 48 hours after birth. The 0–6 month Mother-baby Interaction Observation scale [24] was adapted to rate the efficacy of the intervention. Domains related to eye contact, attention to the baby, reaction to the child's crying and sensitivity and physical contact with babies were recorded. Physical contact with babies, playing with the baby, holding and massaging, and cuddling the baby were also observed and rated. Significant differences and more favourable results in relation to eye contact with the baby, attention and sensitivity reactions were observed in the intervention groups (p < 0.01).

Interventions to improve quality and accessibility of postnatal care for women with disability and their families: In two randomized controlled studies (Keltner et al., Scafidi & Field) [20,22] the intervention was administrated postnatally. The effectiveness of a home visiting program involving weekly visits for a period of one year to promote child-mother interactions was evaluated in 40 women with developmental disability (IQ < 85), and their families in Keltner et al. [20]. Whereas Scafidi & Field [22], explored the effect of massage therapy on the behaviours and weight of 28 neonates born to HIV-positive mothers.

The family configurations in Keltner et al. [20] included women with developmental disability and IQ between 36 and 84 based on scoring on the Slosson Intelligence Test-Revised (SIT-R) [25], and their 12–36 month old children. The intervention was an intense one year family support programme, entitled Supports to Access Rural Services (STARS). Participants were divided into small groups of three to four mothers and their children which met weekly with a family service worker in the community. The programme had three main components: staff training, development of the STARS activities and individual case co-ordination. The programme focused on interpersonal skills, providing information about disability, recognition of health and social barriers, intervention when crisis occurred, cultural sensitivity, community liaison skills and realistic expectations. The control group received a monthly phone call for 12 months, face-to-face assessments every 6 months and appropriate referrals if there was a need.

The effects of a family intervention programme on the maternal-child interaction measures were assessed by Nursing Child Assessment Teaching Scale (NCATS) [26] based on a yes/no 73-point scale. Four subscales focused on parents and two on the child. During the teaching interaction, parents were asked to perform an activity which was about 1.5 months beyond the expected ability of the child. All mothers in the intervention group showed a significant increase in NCATS scores in comparison to baseline: 8.3 for the STARS group and 0.4 for the control group (p < 0.05). Two mothers in the intervention group did not show significant changes and were diagnosed with depression. (See results in Table 1).

Scafidi & Field [22] enrolled 28 singleton neonates born to HIV-positive mothers and assigned them to massage therapy and control groups for a 10-day study duration. Both groups received standard care and were bottled fed without supplements in the neonatal care unit. The massage therapy was administrated to the intervention group for three 15-minute periods during three consecutive hours every day for 10 days (Monday to Friday). The first and third sessions were tactile and the second was kinaesthetic stimulation.

The Brazelton Neonatal Behaviour Assessment Scale-Kanas Supplement [27] was applied to measure the effects of the intervention at baseline (day 1) and post-intervention at day ten. The infants' performance was scored based on 7 factors: habituation, orientation, motor behaviour, range of state, regulation of state, autonomic stability and abnormal reflexes. The massage therapy group performed significantly better on motor, autonomic stability and stress behaviours (p < 0.01). A significant increase in weight gain resulted in the intervention group (p = 0.01).

## Discussion

The literature on interventions targeting disability in pregnancy and postnatally is minimal, with only scarce evidence to inform and promote good practice. Only three studies, all with small sample sizes, were included in this review. All of the interventions reviewed were aimed at women with disability and their families. One study involved delivering training to healthcare professionals, as well as enrolling women with disability. Each of the studies reported that the interventions had a significant positive effect. Two studies were conducted in a hospital setting, giving tighter control over the intervention environment. However, the studies evaluated the effect of the intervention on a relatively small number of participants. The methodological quality of the included studies was moderate to low, with limited information available to assess other biases. We adopted a broad research focus, in terms of types of disability, health care interventions and their timing (during pregnancy, birth and postnatal period), and research participants (women with disability, families and health care professionals). The considerable heterogeneity across the studies was predictable and no meta-analysis could be conducted.

The review was comprehensive in its scope: over 20,000 published papers were identified. However, only a small number of studies fulfilled all of our inclusion criteria. We found that most published papers on disability and pregnancy focused on the underlying medical condition causing the impairment, which was not within the scope of this review. Those which did focus on women with disability were usually case reports, qualitative studies of patient experiences of care or small descriptive studies which were not within scope. Very few were intervention studies and of those that were, most were uncontrolled. Thus, although we identified a large body of literature dealing with the clinical management of specific conditions which are often associated with disability in pregnancy and the postnatal period the published literature on controlled evaluations of interventions to improve maternity care for disabled women in general is extremely sparse.

However, we did find three useful studies which we now consider in context. The one year family home intervention programme demonstrated significant success in improving maternal-child interaction for mothers with developmental disability in Keltner et al. [20]. This study had a reasonable duration but a small sample size. This result is important in overcoming preconceived ideas about the parenting abilities of women with developmental disabilities. This study also highlighted the negative effects of low socio-economic status, low educational achievement, and isolation from society on parenting abilities. Similar findings were reported from a study carried out using national cohort study in the UK in 2000–2002 [3]. However, the sample in the Keltner et al. [20] study was not homogenous in their abilities as participant IQ ranged from 36 to 84.

Two studies (Barbosa Regia, Scafidi & Field) [21,22] in this review enrolled babies of HIV positive mothers, a group considered to be disabled [28] in the UK. Babies born to HIV-positive mothers can be infected with HIV during pregnancy, during labour and delivery, or postpartum via breastfeeding, hence HIV-positive women are advised not to breast feed their babies. HIV positive infants demonstrate less secure attachments than HIV-negative babies [29]. In the study by Scafidi & Field [22] the effectiveness of a massage therapy in improving behaviours and increasing weight of neonates born to HIV-positive mothers was tested. This study had a small sample size of 28 newborns and a short follow-up of only 10 days. The underlying mechanism of weight gain and improved performance with the massaging is unclear. A follow-up study with longer duration is needed to document whether there is a long lasting positive effect of massage. Results from the second study on HIV-positive women and their babies demonstrated the beneficial of an educational video to promote mother-child attachment.

Each of the three randomised trials [20-22] had very small sample sizes (40, 24 and 28 participants respectively) thus we would view their results as promising rather than definitive and suggest the need for further work in these areas, including larger randomised trials.

Our findings are consistent with a recently published review [17] of barriers to accessing health services for women with different types of disability, which concluded that more research in this area is urgently needed. This same review [17] included 87 papers related to barriers and facilitators to access services for women with different types of disability. Most of these studies were of qualitative design, and they were conducted in the UK, Australia, Canada and the US. The studies were grouped into five categories: availability, accessibility, accommodation, affordability and acceptability of the health services. For each form of disability, the various obstacles were listed against each of these five components.

We are unable to draw firm conclusions on which interventions are most effective in improving healthcare outcomes, as sufficient data on outcomes for pregnant women with disability are not yet available. The paucity of evidence is due to a lack of studies on pregnant women with disability in general. Well designed, sufficiently powered intervention studies are required.

The studies currently being reviewed provide very little practical evidence of the efficacy of any interventions. The three RCTs were of a small sample size and were also prone to methodological bias. However, we are able to make some recommendations. It is quite clear that comprehensive support should be routinely provided to the families of women with developmental disabilities. Elsewhere, massage therapy could improve the behaviours and weight of newborn babies to HIV-positive mothers. Overall, HIV-positive women and their babies could benefit from an educational video to promote their attachment. The effectiveness of these interventions and other potential interventions could be established with further research. Another issue that needs to be addressed is the general lack of involvement of women with disabilities in population based research. It may reflect the more general issue that people with disabilities are often excluded from such research.

## Conclusions

The findings of this review indicate substantial gaps in the evidence on evaluated interventions designed to improve outcomes for pregnant women with disability and their families. There is an urgent need to test and evaluate the efficacy of feasible interventions, in addition to further developing study designs for the evaluation of health care interventions targeting women with disability, their families, and healthcare professionals. There is a need for large prospective controlled studies of interventions in this population. We would also suggest that any intervention should be developed in collaboration with women with disability. A recent population-based maternity survey in the UK [30] focusing on the experiences of women with different types of disability, identified some important issues and could inform future studies. As is shown by the survey, women with sensory disability were less likely to breast-feed in comparison with women with no disability (69% vs. 79%). Therefore a breast feeding support programme could be a potentially useful intervention. In addition, women with mental health disabilities were more critical about the support they received and about communications with the staff providing care during their pregnancy. Training programmes to improve staff communication skills could also be evaluated in future controlled design studies.

## Competing interests

The authors declare that they have no competing interest.

## Authors’ contributions

RM and RG wrote the protocol, assessed potentially relevant articles for inclusion/exclusion, conducted the methodological quality reviews, tabulated the extracted data and wrote the first draft of the paper. All authors commented on the protocol, were involved in the interpretation of the findings and edited the manuscript. All authors read and approved the final manuscript.

## Pre-publication history

The pre-publication history for this paper can be accessed here:

http://www.biomedcentral.com/1471-2393/14/58/prepub

## Supplementary Material

Additional file 1 Medline search. Click here for file

Additional file 2 Search report. Click here for file

Additional file 3 Excluded studies. Click here for file
