# Supplementary material for: Systematic review of heath care interventions to improve outcomes for women with disability and their family during pregnancy, birth and postnatal period
Source: BMC Pregnancy Childbirth. 2014 Feb 5;14:58. doi: 10.1186/1471-2393-14-58 (PMC3922586; doi:10.1186/1471-2393-14-58)
Supplement: Additional file 1 — Medline search. [file 1471-2393-14-58-S1.docx]

**Additioanl file 1**

**Medline search:**

1. disabled persons/ or amputees/

2. mobility limitation/

3. Spinal Cord Injuries/

4. paralysis/ or paraplegia/ or quadriplegia/

5. (disabled or disability).ti.

6. ((disabled or disability) adj5 (woman or women or female*)).ti,ab.

7. (spinal cord injur* or paralysis or paralyzed or paraplegi* or tetraplegi* or quadraplegi*).ti,ab.

8. ((physical or physically) adj (disabilit* or disabled)).ti.

9. exp Spinal Dysraphism/

10. Cerebral Palsy/

11. arthritis, rheumatoid/ or arthritis, juvenile rheumatoid/

12. exp Back Pain/

13. spina bifida.ti,ab.

14. cerebral palsy.ti,ab.

15. arthritis.ti,ab.

16. (back adj (pain or ache)).ti,ab.

17. or/1-16

18. vision disorders/ or blindness/

19. hearing disorders/ or hearing loss/ or exp deafness/

20. hearing impaired persons/ or visually impaired persons/

21. (blind or blindness or visually impaired or visual impairment or vision disorder*).ti,ab.

22. (deaf or deafness or hearing impair* or hearing loss).ti,ab.

23. (sensory disabil* or sensory impairment).ti,ab.

24. or/18-23

25. Mentally Ill Persons/

26. Mental Disorders/

27. *Anxiety Disorders/

28. exp affective disorders, psychotic/ or depressive disorder, major/

29. exp "schizophrenia and disorders with psychotic features"/

30. exp Eating Disorders/

31. ((serious or sever* or major or chronic or longterm or long-term or persistent) adj3 (mentally ill or mental illness or mental disorder* or mental health)).ti,ab.

32. (manic* adj depress*).ti,ab.

33. ((serious or sever* or major or chronic or longterm or long-term or persistent) adj3 depress*).ti,ab.

34. ((bipolar or bi-polar) adj3 (depress* or disorder*)).ti,ab.

35. (schizophrenia or schizophrenic or psychos?s or psychotic).ti,ab.

36. (eating disorder* or disordered eating or anorexia nervosa or bulimia or binge eating).ti,ab.

37. or/25-36

38. exp Multiple Sclerosis/

39. exp Muscular Dystrophies/

40. Peripheral Nervous System Diseases/

41. Stroke/

42. Epilepsy/

43. Migraine Disorders/

44. (multiple sclerosis or muscular dystroph*).ti,ab.

45. peripheral neuropath*.ti,ab.

46. stroke.ti,ab.

47. (epilepsy or epileptic).ti,ab.

48. (migraine* or chronic headache*).ti,ab.

49. or/38-48

50. exp Learning Disorders/

51. mental retardation/ or down syndrome/

52. Mentally Disabled Persons/

53. ((learning or intellectual) adj3 (disabilit* or handicap*)).ti,ab.

54. mental retardation.ti,ab.

55. 50 or 51 or 52 or 53 or 54

56. 17 or 24 or 37 or 49 or 55

57. Maternal Health Services/

58. *perinatal care/ or *postnatal care/ or *preconception care/ or *prenatal care/

59. Pregnant Women/

60. exp Pregnancy/

61. (pregnant or pregnancy).ti,ab.

62. ((maternal health or prenatal or pre-natal or prepartum or pre-partum or antenatal or ante-natal or perinatal or postnatal or post-natal or postpartum or post-partum or pueprium or puepral) adj3 (care or service* or healthcare)).ti,ab.

63. 57 or 58 or 59 or 60 or 61 or 62

64. 56 and 63

65. limit 64 to (comment or editorial or letter or meta analysis or "review")

66. exp animals/ not human/

67. 64 not (65 or 66)

68. randomized controlled trial.pt.

69. controlled clinical trial.pt.

70. randomized.ab.

71. placebo.ab.

72. drug therapy.fs.

73. randomly.ab.

74. trial.ab.

75. groups.ab.

76. 68 or 69 or 70 or 71 or 72 or 73 or 74 or 75

77. 67 and 76

78. intervention*.ti.

79. (intervention* adj6 (clinician* or collaborat* or community or complex or DESIGN* or doctor* or educational or family doctor* or family physician* or family practitioner* or financial or GP or general practice* or hospital* or impact* or improv* or individuali?e* or individuali?ing or interdisciplin* or multicomponent or multi-component or multidisciplin* or multi-disciplin* or multifacet* or multi-facet* or multimodal* or multi-modal* or personali?e* or personali?ing or pharmacies or pharmacist* or pharmacy or physician* or practitioner* or prescrib* or prescription* or primary care or professional* or provider* or regulatory or regulatory or tailor* or target* or team* or usual care)).ab.

80. (collaborativ* or collaboration* or tailored or personali?ed).ti,ab.

81. (exp hospitals/ or exp Hospitalization/ or exp Patients/ or exp Nurses/ or exp Nursing/) and (study.ti. or evaluation studies as topic/)

82. demonstration project*.ti,ab.

83. (pre-post or "pre test*" or pretest* or posttest* or "post test*" or (pre adj5 post)).ti,ab.

84. (pre-workshop or post-workshop or (before adj3 workshop) or (after adj3 workshop)).ti,ab.

85. ((study adj3 aim?) or "our study").ab.

86. (before adj10 (after or during)).ti,ab.

87. ("quasi-experiment*" or quasiexperiment* or "quasi random*" or quasirandom* or "quasi control*" or quasicontrol* or ((quasi* or experimental) adj3 (method* or study or trial or design*))).ti,ab,hw.

88. ("time series" adj2 interrupt*).ti,ab,hw.

89. (time points adj3 (over or multiple or three or four or five or six or seven or eight or nine or ten or eleven or twelve or month* or hour? or day? or "more than")).ab.

90. pilot.ti.

91. Pilot projects/

92. clinical trial.pt.

93. multicenter study.pt.

94. (multicentre or multicenter or multi-centre or multi-center).ti.

95. random*.ti,ab. or controlled.ti.

96. (control adj3 (area or cohort? or compar? or condition or group? or intervention? or participant? or study)).ab.

97. 78 or 79 or 80 or 81 or 82 or 83 or 84 or 85 or 86 or 87 or 88 or 89 or 90 or 91 or 92 or 93 or 94 or 95 or 96

98. 67 and 97

99. 77 or 98
