# Supplementary material for: Systematic review of heath care interventions to improve outcomes for women with disability and their family during pregnancy, birth and postnatal period
Source: BMC Pregnancy Childbirth. 2014 Feb 5;14:58. doi: 10.1186/1471-2393-14-58 (PMC3922586; doi:10.1186/1471-2393-14-58)
Supplement: Additional file 2 — Search report. [file 1471-2393-14-58-S2.docx]

**Additioanl file 2**

Search report

| **Database name:** | **Interface:** | **Year range:** | **Date searched:** | **Hits:** |
| --- | --- | --- | --- | --- |
| British Nursing Index CINAHL Cochrane Central Register of Controlled Trials Dissertations & Theses A&I Embase Medline PsycINFO Social Science Citation Index Sociological Abstracts | Proquest EbscoHOST Wiley Proquest OvidSP OvidSP OvidSP WoK Proquest | 1994 – 1980 - Issue 2, 2012 1639 - 1974 – 1946 – 1967 - 1945 - 1952 - | 09/03/2012 09/03/2012 12/03/2012 12/03/2012 09/03/2012 09/03/2012 09/03/2012 12/03/2012 12/03/2012 | 360 1439 4551 99 7205 9191 2582 3085 406 |
| Total number of records retrieved =  28918 Number of duplicates removed =  7799 Final total =  21119 | | | | |
| **Limits:**  Language: Non-applied Human: animal studies excluded Publication type: RCT filter and EPOC methodological filters applied where possible. Comment, editorials, letters and reviews excluded. | | | | |
