# Supplementary material for: Systematic review of heath care interventions to improve outcomes for women with disability and their family during pregnancy, birth and postnatal period
Source: BMC Pregnancy Childbirth. 2014 Feb 5;14:58. doi: 10.1186/1471-2393-14-58 (PMC3922586; doi:10.1186/1471-2393-14-58)
Supplement: Additional file 3 — Excluded studies. [file 1471-2393-14-58-S3.docx]

**Additional file 3**

| **Study** | **Reasons for exclusion** |
| --- | --- |
| 1. **Ajh 2006** | Randomized controlled trial (RCT), the aim of the study is to examine the effect of the intervention on the post natal depression, not disabled women. |
| 1. **Alakus 2007** | Report of 34 clinicians from mental health services to explore their views on services provided to parents with mental health problems with children less than 5 years of age, not an EPOC design. |
| 1. **Alder 2011** | RCT included women with anxiety related pregnancy, not disabled. |
| 1. **Ammaniti 2006** | RCT evaluated the effect of an early home-visiting intervention in mothers at risk of developing depression, not disabled women. |
| 1. **Ane 2006** | Report, not an EPOC design. |
| 1. **Baker 2003** | A case report; describes the reality of childbirth and child-rearing for women with thalidomide teratogenic effects and the help from an occupational therapist through newborn, bathing, dressing, feeding and other needs for new baby, not an EPOC design. |
| 1. **Bennedsen 2001** | Cohort study comparing complications during pregnancy of women with and without schizophrenia, no intervention. |
| 1. **Blackford 2000** | Descriptive semi structured interviews, not an EPOC design. |
| 1. **Buka 2000** | Cohort study comparing the maternal recalling between mothers of offspring with psychosis and mothers of offspring with no psychosis not disabled women. |
| 1. **Calderon-Margalit 2009** | A cohort study involving 2793 pregnant women looking at the association of maternal psychotropic medication use and adverse pregnancy outcomes, this is not the focus of this review. |
| 1. **Carter 2003** | A follow-up of a randomised controlled trial to assess the effect of cognitive behaviour therapy on the symptoms of bulimia nervosa, not disabling women. |
| 1. **Carty 1998** | Case report, not an EPOC design. |
| 1. **Cantrell 2009** | Case report, not an EPOC design. |
| 1. **Chevarley 2006** | Health interview survey, not an EPOC design |
| 1. **Clinton 1988** | Participants were low income families, not disabled women. |
| 1. **Coverdale 2004** | Review, not an EPOC design. |
| 1. **Creighton C** | Case report, not an EPOC design. |
| 1. **Crncec 2009** | Mothers with postnatal depression, not disabled women. |
| 1. **Dudzinski 2004** | Case report, not an EPOC design. |
| 1. **Duran 2008** | Case report, not an EPOC design. |
| 1. **Earle 2004** | Report, not an EPOC design. |
| 1. **Einarson 2001** | Counselling services involving not disabled women. |
| 1. **Feinberg 2010** | RCT, problem-solve education program for women at risk of developing depression. |
| 1. **Frieder 2008** | Report, not an EPOC design. |
| 1. **Galbally 2010** | Report, not an EPOC design. |
| 1. **Gavin 2006** | Cohort study with no evaluation of an intervention. |
| 1. **Geddes 1999** | Meta-analysis of the association between complications of pregnancy and delivery and schizophrenia, no intervention. |
| 1. **Goodman 1994** | Survey design study, not an EPOC design. |
| 1. **Hauck 2008** | Developing a case management framework for women with mental health problems, not an EPOC design and no evaluation to the intervention. |
| 1. **Hellwig 2007** | Observational study 73 women with multiple sclerosis (MS) with 88, no intervention. |
| 1. **Howard 2003** | Cohort study to show the rate of still birth, neonatal death and gestational age in women with psychotic disorders, no intervention. |
| 1. **Howard 2007** | Study assessed the validity of Camberwell Assessment of Need-Mother version (CAN, M) for pregnant women with severe mental illness, no intervention. |
| 1. **Jablensky 2005** | Population cohort study to illustrate the complications during pregnancy, delivery and neonatal in women with schizophrenia and major disorders, no intervention. |
| 1. **Jackson 1999** | Cross-sectional survey, women with spinal cord injuries reporting their reproductive issues in relation to their disability, no intervention. |
| 1. **Jones 2007** | Quasi-experimental, pre- post-test study; 84 deaf not pregnant, no intervention. |
| 1. **Kalinauskiene 2009** | Randomised controlled trial testing the effect of video-feedback on mother's sensitivity, which is not a disabling disorder. |
| 1. **Kalus 2007** | Randomised controlled trial; involving women with back pain related to pregnancy, not disabled women. |
| 1. **Lewis 2006** | Cohort study; mothers to children age 8 to 12 years with breast cancer not pregnant. |
| 1. **Lin 2009** | Comparing the number of antenatal visits between women with or without schizophrenia, no intervention. |
| 1. **MacArthur 2002** | Cluster randomised controlled trial; postnatal model care for women, not women with disability. |
| 1. **MacCabe 2007** | Nested controlled study to compare preterm birth, stillbirth, low birth weight, gestational age of mothers with psychosis to normal mothers, no intervention. |
| 1. **McCauley-Elsom 2007** | Case report, not an EPOC design. |
| 1. **McCullough 2002** | Review, not an EPOC design |
| 1. **McGuire 2003** | Case report, not an EPOC design. |
| 1. **Moore 1997** | Report on improving health access for disabled, not an EPOC design. |
| 1. **NDA 2001** | Social survey interviews, not an EPOC design. |
| 1. **NDA 2007** | Social survey interviews, not an EPOC design. |
| 1. **NDA 2011** | Social survey interview, not an EPOC design. |
| 1. **Nishizawa 2007** | Providing care and support of schizophrenic women during pregnancy in order to stabilize the symptoms, this is not the focus of this review. |
| 1. **Penidl 2007** | Cohort study, no intervention. |
| 1. **Prunty 2007** | Randomised controlled trial, including women with multiple sclerosis not pregnant. |
| 1. **Robinson-Whelen 2007** | Randomised controlled trial, including women with disabilities and depressed, but not pregnant. |
| 1. **Rogers 2005** | Qualitative study, not an EPOC design. |
| 1. **Sandford 2003** | Randomized controlled trail, parents with depression (not pregnant mothers) with older 6-12 years children. |
| 1. **Smeltzer 2006** | Report, not an EPOC design. |
| 1. **Stein 2006** | Randomized controlled trial, mothers with eating disorders in the postnatal period not disabled. |
| 1. **Thorne 1990** | A qualitative study, including 77 participants, 16 were mothers with chronic illness, not an EPOC design. |
| 1. **Underwood 2004** | A case report of a deaf mother, not an EPOC design. |
| 1. **Warner 2001** | Review of interventions to prevent schizophrenia in obstetrical care settings. |
| 1. **Wong 2000** | A semi-structured interview of women with physical disabilities, not an EPOC design. |
| 1. **Zhydkova 2009** | Observational controlled study involving women with polycystic ovary syndrome (PCOS), not disabled women. |

**References:**

1. Ajh N Unesian: **The study of supportive activities during pregnancy on postpartum depression [Farsi].** *Hayat* 2006, **12**(3):88.

2. Alakus C, Conwell R, Gilbert M, Buist A, Castle D: **The needs of parents with a mental illness who have young children: an Australian perspective on service delivery options.** *International Journal of Psychiatry* 2007, **53**:333.

3. Alder J, Urech C, Fink N, Bitzer J, Hoesli I**: Response to induced relaxation during pregnancy: Comparison of women with high versus low levels of anxiety**. *Journal of Clinical Psychology Med Settings* 2011, **18**:13-21.

4. Ammaniti M, Speranza AM, Tambelli R: **A prevention and promotion intervention program in the field of mother-infant relationship.** *Infant Mental Health Journal* 2006, **27** (1): 70-90.

5. Ane A M: "**Maternity support care of deaf women**. [French]." *Soins Pediatrie/Puericulture* 2006 **27**(229): 29-30.

6. Baker S: **Beating disability, embracing motherhood**. *The Practicing Midwife* 2003, **6**:16-17.

7. Bennedsen BE, Mortensen PB, Olesen AV, Henriksen TB**: Obstetric complications in women with schizophrenia.** *Schizophrenia Research* 2001, **47**:167-175.

8. Blackford KA: **Prenatal education for mothers with disability**. *Journal of Advanced Nursing 2000,* 32, **4**: 898-904.

9. Buka S, Goldstein JM, Seidman LJ, Tsuang MT**: Maternal recall of pregnancy history: Accuracy and bias in schizophrenia research.** Schizophrenia Bulletin 2000, **26** (2):335-350.

10. Calderon-Margalit RC, Qiu C, Ornoy A, Siscovick DS, Williams MA: **Risk of preterm delivery and other adverse perinatal outcomes in relation to maternal use of psychotropic medications during pregnancy**. *American Journal of Obstetrics & Gynecology* 2009, **201**(6): 571-578.

11. Carter FA, McIntosh VV, Joyce PR, Frampton CM, Bulik CM: **Bulimia nervosa, childbirth and psychopathology.** *Journal of Psychosomatic Research* 2003, **55** (4): 357-361.

12. Carty E: **Disability and childbirth: meeting the challenges.**  *Canadian Medical Association* 1998:363-369.

13. Cantrell CT, Kelley T, McDermott T: **Midwifery management of the women with an eating disorder in the antepartum period.** *Journal of Midwifery and Women's Health* 2009, **54** (6): 503-508.

14. Chevarley F, Thierry J, Gill C, Ryerson B, Nosek M**: Health preventive care and health care access among women with disabilities in the 1994-1995 national health interview survey supplement on disability**. *Women’s Health Issues* 2006, **16**: 297-312.

15. Clinton B, Larner M: **Rural community women as leaders in health outreach**. *Journal of Primary Prevention* 1988, **9**(1&2):120-129.

16. Coverdale JH, McCullough LB, Chervenak FA: **Assisted and Surrogate Decision Making for Pregnant Patients Who Have Schizophrenia.** *Schizophrenia Bulletin* 2004, **30** (3):659-64.

17. Creighton C**: Pregnancy and quadriplegia: An occupational therapy home program**. *The American Journal of Occupational Therapy* 1989, **1**: 44-46.

18. Crncec R, Cooper E, Stephen M: **Treating infant sleep disturbance: Does maternal mod impact upon effectiveness?** *Journal of Pediatrics and Child Health* 2010, **46**:29-34.

19. Dudzinski DM, Sullivan M**: When agreeing with the patient is not enough: a schizophrenic woman requests pregnancy termination**. *General Hospital Psychiatry* 2004, **26**:475-480.

20. Duran AM, Ugur M**: Clozapine use in two women with schizophrenia during pregnancy*.*** *Journal of Psychopharmacology* 2008, **22**(1):111-13.

21. Earle S, Church S**: Disability and reproduction**. *The practicing Midwife* 2004, **7** (8):32-34.

22. Einarson A, Selby P, Koren G: **Abrupt discontinuation of psychotropic drugs during pregnancy: fear of teratogenic risk and impact of counselling**. *Journal of Psychiatry and Neuroscience* 2001,**1**: 44-8.

23. Feinberg E: **Reducing Risk After an Adverse Pregnancy Outcome.** 2011, Clinical trials.gov available at : <http://clinicaltrials.gov/ct2/show/NCT01182363?term=pregnancy+and+disability&rank=17>.

24. Frieder A, Dunlop AL, Culpepper L, Bernstein PS: **The clinical content of preconception care: women with psychiatric conditions.** *American Journal of Obstetrics and Gynecology* 2008, Supplement to December: S328-S332.

25. Galbally M, Snellen M: **Management of antipsychotic and mood stabilizer medication in pregnancy: recommendations for antenatal care**. *Australian & New Zealand Journal of Psychiatry* 2010, **44**, 2:99-108.

26. Gavin NI, Bendecit B, Adams KE**: Health service use and outcomes among disabled medical pregnant women.** Women's Health Issues 2006, **16**:313-322.

27. Geddes JR, Verdoux H: **Schizophrenia and complications of pregnancy and labour: an individual patient data meta-analysis.** *Schizophrenia Bulletin* 1999, **25**, 3:413-23.

28. Goodman M: **Pregnant and disabled? Don't assume the professionals will understand**. *Professional care of Mother and Child* 1994, **4** (8):227-228.

29. Hauck Y, Rock D, Jackiewicz, T, Jablensky A : **Health babies for mothers with serious mental illness: A case management framework for mental health clinicians.** *International Journal of Mental Health Nursing* 2008, **17**:383-391.

30. Hellwig K, Brune N, Haghikia A, Muller T, Schimrigk S, Schwodiauer V, Gold R: **Reproductive counseling, treatment and course of pregnancy in 73 German MS patients.** *Acta Neurologica Scandinavica* 2008, **118** (1):24-28.

31. Howard LM, Goss C, Leese M, Thornicroft G: **Medical outcome of pregnancy in women with psychotic disorders and their infants in the first year after birth**. *British Journal Of Psychiatry* 2003, **182**:63-67.

32. Howard LK, Hunt K, Slade M: **Assessing the needs of pregnant women and mothers with severe mental illness: the psychometric properties of the Camberwell Assessment of Need - Mothers (CAN-M).**" *International Journal of Methods in Psychiatric Research* 2007, **16**, (4):177-85.

33. Jablensky AV, Morgan V**: Pregnancy, delivery, and neonatal complications in a population cohort of women with schizophrenia and major affective disorders**. *American Journal of Psychiatry* 2005, **162** (1):79-91.

34. Jackson AB, Wadley VA: **Multicenter Study of Women's Self-Reported Reproductive Health After Spinal Cord Injury**. *Archives of Physical Medicine and Rehabilitation* 1999, **80**:1420-8.

35. Jones EG, Renger R, Kang Y**: Self-efficacy for health-related behaviours among deaf adults.** *Research in Nursing and Health* 2007, **30**:185-192.

36. Kalinauskiene L, Cekuoliene D, Van Ijzendoorn MH, Bakermans-Kranenburg MJ, Juffer F, Kusakovskaja I: **Supporting insensitive mothers: the Vilnius randomized control trial of video-feedback intervention to promote maternal sensitivity and infant attachment security**. *Child: Care, Health and Development* 2009, **35**(5):613-23.

37. Kalus SM, Kornman LH, Quinlivan JA: **Managing back pain in pregnancy using a support garment: a randomised trial.** *International Journal of Obstetrics and Gynaecology* 2008, **115**:68-75.

38. Lewis FM, Casey SM, Brandt PA, Shands ME, Zahli EH: **The enhancing connections program: pilot study of a cognitive-behavioral intervention for mothers and children affected by breast cancer**. *Psycho-Oncology* 2006, **15**(6):486-97.

39. Lin HC, Chen YH, Lee HC: **Prenatal care and adverse pregnancy outcomes among women with schizophrenia; a national population-based study in Taiwan**. *Journal of clinical Psychiatry* 2009, **70** (9):1297-1303.

40. MacArthur C, Winter HR, Bick DE, Knowlers H, Lilford R, Henderson C, Lancashire RJ, Braunholtz DA, Gee H: **Effects of redesigned community postnatal care on women's health 4 months after birth: a cluster randomised controlled trial.** *The Lancet* 2002, **359**:378-85.

41. MacCabe JH, Martinsson L: **Adverse pregnancy outcomes in mothers with affective psychosis.** *Bipolar Disorders* 2007, **9** (3):305-309.

42. McCauley-Elsom K, Kulkarni J: **Managing psychosis in pregnancy.** Australian & New Zealand Journal of Psychiatry 2007, **41** (3):289-292.

43. McCulough LB, Coverdale JH, Chervenak FA: **Ethical challenges of decision making with pregnant patients who have schizophrenia.** *American Journal of Obstetrics and Gynecology* 2002, **187**(3):696-702.

44. McGuire K: **Second-class services: a visually impaired mother's story.** *The Practicing Midwife* 2003, **6**(7):18-20.

45. Moore G: **"Improving health access: it’s about attitude**".. *Nursing BC* 1997, **29** (3):27-30.

46. National Disability Authority (NDA) 2001: Public attitudes to disability in the republic of Ireland 2001. Available at: <http://www.nda.ie/cntmgmtnew.nsf/0/910C72601D161DE580256CCC005373D5?OpenDocument>

47. National Disability Authority (NDA) 2006: Public attitudes to disability in Ireland 2006. Available at: <http://www.nda.ie/cntmgmtnew.nsf/0/FD9B9DBF1F1CF617802573B8005DDED5?OpenDocument>

48. National Disability Authority (NDA) 20111: National survey of public attitudes to disability in Ireland 2011. Available at: <http://www.nda.ie/website/nda/cntmgmtnew.nsf/0/90F8D23334D786A880257987004FCF51?OpenDocument>

49. Nishizawa O, Sakumoto K: **Effectiveness of comprehensive supports for schizophrenic women during pregnancy and puerperium: preliminary study**. *Psychiatry & Clinical Neurosciences* 2007, **61**(6):665-671.

50. [Peindl KS](http://www.ncbi.nlm.nih.gov/pubmed?term=Peindl%20KS%5BAuthor%5D&cauthor=true&cauthor_uid=18032983), [Masand P](http://www.ncbi.nlm.nih.gov/pubmed?term=Masand%20P%5BAuthor%5D&cauthor=true&cauthor_uid=18032983), [Mannelli P](http://www.ncbi.nlm.nih.gov/pubmed?term=Mannelli%20P%5BAuthor%5D&cauthor=true&cauthor_uid=18032983), [Narasimhan M](http://www.ncbi.nlm.nih.gov/pubmed?term=Narasimhan%20M%5BAuthor%5D&cauthor=true&cauthor_uid=18032983), [Patkar A](http://www.ncbi.nlm.nih.gov/pubmed?term=Patkar%20A%5BAuthor%5D&cauthor=true&cauthor_uid=18032983)**:Polypharmacy in pregnant women with major psychiatric illness: a pilot study.** [*J Psychiatr Pract*](http://www.ncbi.nlm.nih.gov/pubmed/18032983) 2007 Nov 13, **6**: 385-92.

51. Prunty MC, Sharpe L, Butow Ph, Fulcher G. **The motherhood choice: A decision aid for women with multiple sclerosis.** *Patient Education and Counseling* 2008, **71**:108-115.

52. Robinson-Whelen S, Hughes RB, Taylor HB, Hall JW, Rehm LP**: Depression Self-Management Program for Rural Women With Physical Disabilities.** *Rehabilitation Psychology* 2007, 52, 3:254-262.

53. Rogers J, Matsumura M: **The Disabled Woman’s Guide to Pregnancy and Birth**. Demos Medical publishing 2005.

54. Sanford MC, Byrne C, Williams S, Atley S, Ridley T, Miller J, Allin H: **A pilot study of a parent-education group for families affected by depression.** *Canadian Journal of Psychiatry - Revue Canadienne de Psychiatrie* 2003, **2**:78-86.

55. Smelzer SC: **Pregnancy in women with physical disabilities**. *Journal of Obstetric, Gynecologic, and Neonatal Nursing* 2006, **36** (1):88-96.

56. Stein A, Woolley H, Senior R, Hertzmann L, Lovel M, Lee J, Cooper S, Wheatcroft R, Challacombe F, Patel P, Nicol-Harper R, Menzes P, Schmidt A, Juszczak E, Fairburn Ch: **Treating disturbances in the relationship between mothers with bulimic eating disorders and their infants: A randomized, controlled trial of video feedback.** *American Journal of Psychiatry* 2006, **163**:899-906.

57. Thorne S: **Mothers with chronic illness: A predicament of social construction.** *Health Care for Women International* 1990, **11** (2):209-221.

58. Underwood Ch:**Maternity services are failing deaf women**. *Journal of Family Health Care* 2004, **14**(2):30-31.

59. Warner R: **The prevention of schizophrenia: What interventions are safe and effective?** *Schizophrenia Bulletin* 2001, **27** (4): 551-562.

60. Wong A: **The work of disabled women seeking reproductive health care**. *Sexuality and Disability* 2000, **18** (4):301-306.

61. Zhydkova IA, Karlov VA:**Reproductive potential of women with epilepsy [Zhurnal Nevrologii i Psihiatrii imeni S.S]** *Korsakova* 2009, **109** (11):31-36.
